# Potato precision planter metering system based on improved YOLOv5n-ByteTrack

**DOI:** 10.3389/fpls.2025.1563551

**Published:** 2025-04-28

**Authors:** Cisen Xiao, Changlin Song, Junmin Li, Min Liao, Yongfan Pu, Kun Du

**Affiliations:** ^1^ School of Computer and Software Engineering, Xihua University, Chengdu, China; ^2^ School of Mechanical Engineering, Xihua University, Chengdu, China

**Keywords:** potato, object detection, lightweight, multiple object tracking, YOLOv5n

## Abstract

Accurate assessment of the planting effect is crucial during the potato cultivation process. Currently, manual statistical methods are inefficient and challenging to evaluate in real-time. To address this issue, this study proposes a detection algorithm for the potato planting machine’s seed potato scooping scene, based on an improved lightweight YOLO v5n model. Initially, the C3-Faster module is introduced, which reduces the number of parameters and computational load while maintaining detection accuracy. Subsequently, re-parameterized convolution (RepConv) is incorporated into the feature extraction network architecture, enhancing the model’s inference speed by leveraging the correlation between features. Finally, to further improve the efficiency of the model for mobile applications, layer-adaptive magnitude-based pruning (LAMP) technology is employed to eliminate redundant channels with minimal impact on performance. The experimental results indicate that: 1) The improved YOLOv5n model exhibits a 56.8% reduction in parameters, a 56.1% decrease in giga floating point operations per second (GFLOPs), a 51.4% reduction in model size, and a 37.0% reduction in Embedded Device Inference Time compared to the YOLOv5n model. Additionally, the mean average precision (mAP) at mAP@0.5 achieves up to 98.0%. 2) Compared with the YOLO series model, mAP@0.5 is close, and the parameters, GFLOPs, and model size are significantly decreased. 3) Combining the ByteTrack algorithm and counting method, the accuracy of counting reaches 96.6%. Based on these improvements, we designed a potato precision planter metering system that supports real-time monitoring of omission, replanting, and qualified casting during the planting process. This system provides effective support for potato precision planting and offers a visual representation of the planting outcomes, demonstrating its practical value for the industry.

## 1 Introduction

Potatoes are one of the most widely consumed food crops globally, ranking fourth after maize, wheat, and rice. As a nutrient-rich staple food, potatoes are not only a significant dietary component in many countries but also play a crucial role in global food security and sustainable agricultural development (Dai et al., 2022; Gao et al., 2023). Their high yield potential and adaptability enable growth under various climatic conditions, making them pivotal in addressing global population growth and food scarcity challenges. Furthermore, the short cultivation cycle and minimal soil requirements of potatoes make them a vital choice for poverty alleviation and food insecurity mitigation in numerous developing countries. With the continuous advancement of modern technology, mechanized planting has increasingly become a key technological component of agricultural mechanization, particularly in potato cultivation, where its significance is becoming more pronounced. The application of mechanized planting technologies in potato cultivation has not only significantly enhanced production efficiency and reduced labor intensity but also enabled precise seeding and fertilization, effectively promoting a dual increase in potato yield and quality (Zhou et al., 2022). Nevertheless, in the process of mechanized potato planting, traditional reliance on manual statistical methods for evaluating planting outcomes is not only cumbersome and time-consuming but also inefficient. This challenge is exacerbated in the face of vast planting areas, making it nearly insurmountable. Consequently, the adoption of an efficient and accurate precision planting metering system for potatoes as a substitute for traditional manual methods is of paramount importance for advancing precision potato planting.

In recent years, the continuous advancement of deep learning-based object detection algorithms, such as YOLO (You Only Look Once), has revolutionized various fields, with significant applications in agriculture. Object detection algorithms, particularly YOLO, have become essential tools for the automatic identification and localization of objects in images or videos, offering substantial benefits for tasks that require high precision and real-time performance. In agriculture, these algorithms have garnered attention for applications such as crop detection (Gui et al., 2023; Meng et al., 2023), plant disease identification (Li et al., 2022), fruit quality detection (Karthikeyan et al., 2024), pest monitoring, livestock management, and other critical tasks, with promising results. For example, Rashid et al. (2021) applied the YOLOv5 image segmentation technique to extract potato leaves and detect early and late blight diseases using convolutional neural networks (CNNs). Their proposed multilevel deep learning model for potato leaf disease recognition demonstrated excellent accuracy. Huang et al. (2023a) proposed a lightweight CNN-based model for the accurate and efficient detection of potato seed eyes in automated cutting equipment. By integrating GhostNetV2 as the backbone for YOLOv4, along with depthwise separable convolutions and the SCYLLA-IoU loss function, the model reduced inference parameters while enhancing detection performance. Wang et al. (2024c) utilized VanillaNet as the backbone network and introduced the VBGS-YOLOv8n model for potato seedling detection. They also developed a dataset of potato seedling images captured by drones, providing valuable technical support for monitoring potato health. These advancements underscore the growing importance of object detection algorithms in addressing complex agricultural challenges.

Furthermore, target tracking algorithms have continuously evolved, progressing from SORT (Simple Online and Realtime Tracker) (Bewley et al., 2016) to MOT (Multi-Object Tracking) (Tang et al., 2017; Zhang et al., 2022) and its various variants (Wojke et al., 2017; Chu et al., 2017). MOT plays a crucial role in applications such as crop counting and livestock tracking, where the continuous monitoring of multiple objects is essential. The integration of object detection with multi-object tracking enables effective handling of dynamic scenes involving multiple moving targets. Research has demonstrated the synergy between these two techniques, showcasing their potential to enhance real-time tracking and monitoring applications. For example, Rong et al. proposed an improved tomato cluster counting method by combining object detection, multi-object tracking, and specific tracking region counting. To address background misidentification of tomatoes, they introduced YOLOv5-4D, which fuses RGB and depth images, while ByteTrack was used to track tomato clusters across frames, and a specific tracking region counting method was designed to resolve ID shifts in tracked clusters (Rong et al., 2023). Huang et al. proposed an improved pig counting algorithm (MPC-YD) that integrates YOLOv5 and DeepSORT to overcome challenges such as manual counting inefficiency, rapid movement, and tracking deviations (Huang et al., 2023b). Yang et al. improved the YOLOv5s model to develop an automatic identification and counting system for laying hens (Yang et al., 2023). Similarly, Liu et al. enhanced the YOLOv7 model with the SimAM attention mechanism and integrated the ByteTrack algorithm for a real-time system to count dried Hami jujubes (Liu et al., 2024b), while Huang et al. combined YOLOv5 with DeepSORT for rapeseed seedling detection and counting (Huang et al., 2024). These studies showcase how the integration of object detection with multi-object tracking can address practical challenges in agricultural production.

While substantial progress has been made in the development and application of these technologies, challenges remain, particularly in terms of scalability and robustness under diverse environmental conditions. For example, occlusions, variations in lighting, and different growth stages of crops can hinder the performance of existing algorithms. The current research continues to explore ways to enhance the accuracy, efficiency, and generalization of these models, paving the way for more reliable automation in agriculture. This paper builds upon previous work by applying advanced object detection and multi-object tracking algorithms to the specific problem of detecting and counting potato seed tubers and seed scoops during the planting process.

In this study, the YOLOv5n model was optimized for lightweight performance, with the objective of further diminishing the model’s parameter count and accelerating target detection, thereby enhancing its viability for deployment on computationally restricted embedded devices. Concurrently, the improved YOLO v5n model was integrated with the Bytetrack algorithm, resulting in the development of a novel counting and line-drawing algorithm that achieves precise enumeration of potato seed tubers and seed scoops. Utilizing the QT framework, a precision planting and measurement system for potatoes was constructed. This system, operational on an embedded device, enables real-time detection and enumeration of potato seed tubers and seed scoops during planting, while concurrently harvesting real-time sensor data. The system processes calculations in accordance with established criteria and presents the potato planting outcomes in real-time on the display interface.

The main contributions of this paper can be summarized as follows:

1. A dataset was constructed on potato planters picking up potato seed tubers, which included two types: potato seed tubers and seed scoops.
2. In comparison with the original YOLOv5n algorithm, the improved YOLOv5n version exhibits an improvement of 0.7 percentage points in the mAP@0.5 metric, a reduction of 56.8% in the number of parameters, a decrease of 56.1% in GFLOPs, a 51.4% reduction in model size, and a 37.0% reduction in Embedded Device Inference Time.
3. By integrating the improved YOLOv5n algorithm with the Bytetrack algorithm and designing a counting method, we have successfully achieved precise identification and counting of potato seed tubers and seed scoops. Concurrently, we have developed a precision planting metering system for potatoes based on this technology.

## 2 Materials and methods

### 2.1 Data acquisition

**Figure 1:**
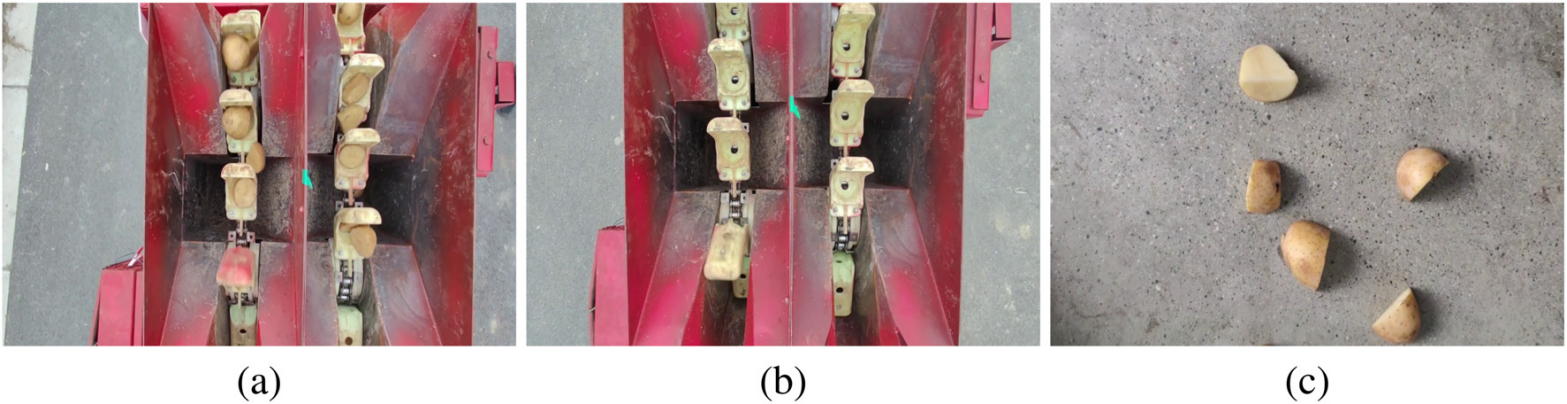
Dataset instance. **(a)** Potato seed tubers and seed scoops; **(b)** Seed scoops; **(c)** Potato seed tubers.

The dataset comprises two categories: potato seed tubers and seed scoops. For ease of integration and development, we use a USB industrial camera (JIERUIWEITONG DF500). This camera supports 30fps and is ideal for many application scenarios due to its easy accessibility, high cost-effectiveness, and strong compatibility, especially in situations where high image acquisition accuracy is required but the budget is limited. In order to make the camera just enough to capture the seed taking process of the potato planter, at the test site of the Institute of Modern Agricultural Equipment Research of Xihua University, we fixed the industrial camera on the bracket above the seed-clearing position of the potato precision planter, and successfully recorded the process of seed retrieval by a potato precision planter as well as the video containing only the seed scoops. In addition, by means of a hand-held industrial camera, we also recorded videos containing only the seed scoops and only the potato seed tubers, totaling 30 video files. To facilitate the extraction of target images from the videos, segments devoid of the target categories were excised. For the edited videos, OpenCV functions were used to extract image frames, which were then saved in a 640×480 pixel RGB format. Ultimately, 1500 images of the potato planter’s seed retrieval process, 400 images of seed scoops, and 800 images of potato seed tubers were obtained, including those with potato seed tubers coated in talc. **Figure 1** displays a selection of the collected image samples.

**Figure 2:**
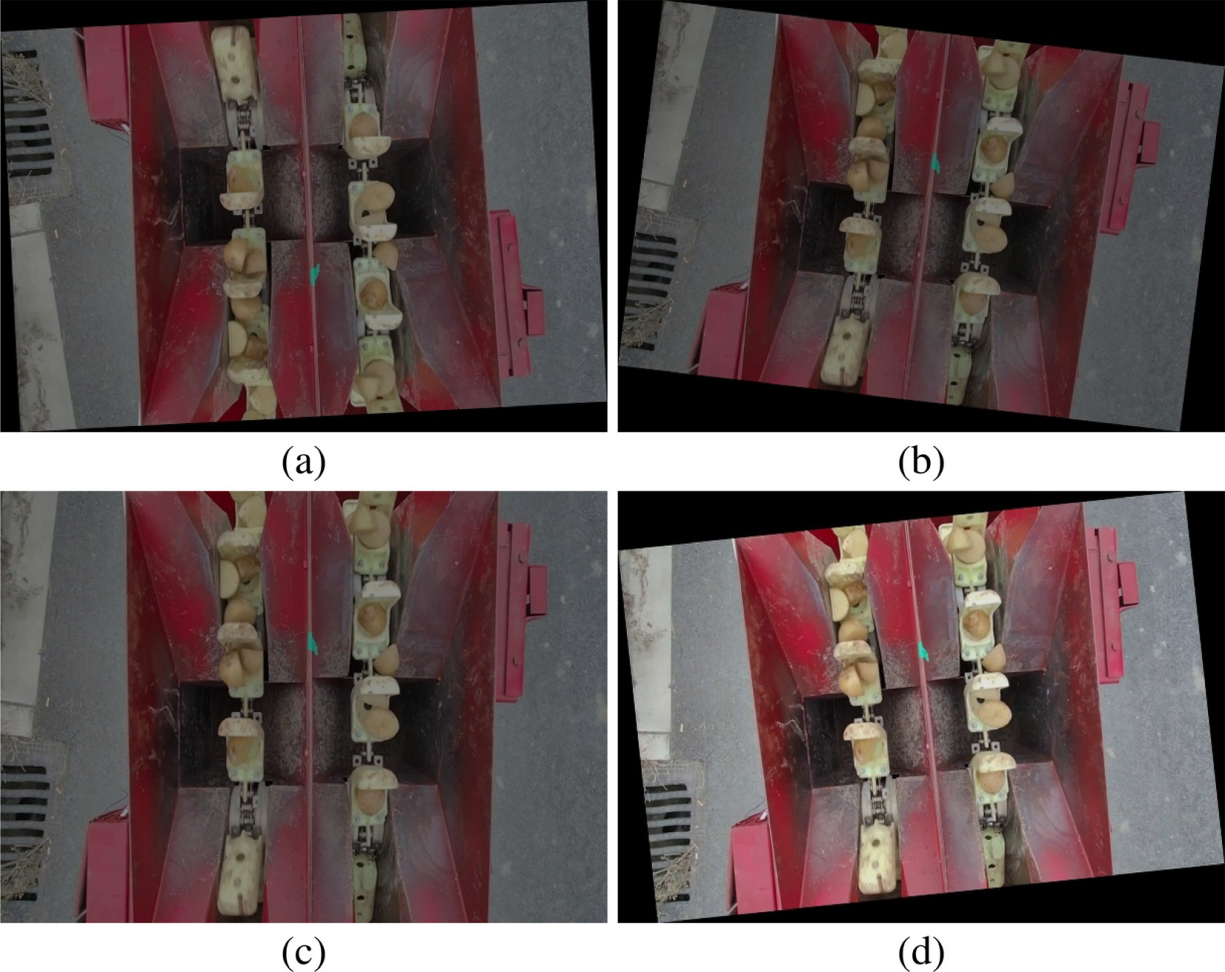
Example of data augmentation. **(a)** The image after undergoing rotation, brightness adjustment, noise addition, and flipping; **(b)** The image after undergoing rotation, brightness adjustment, and noise addition; **(c)** The image after undergoing brightness adjustment and noise addition; **(d)** The image after undergoing rotation.

Utilizing the LabelImg tool, all samples within the image dataset were manually annotated, categorizing potato seed tubers as the “green” class and seed scoops as the “red” class, with corresponding XML formatted label data generated. In order to enhance the diversity of the samples, four data enhancement methods including rotating, adjusting brightness, adding noise and flipping were used to process the image data to ensure that each image was randomly enhanced four times. After the data augmentation process, a total of 13,500 image samples were obtained, including the original images. The enhancement effect of some samples is shown in **Figure 2**. To maintain the randomness of the experimental data, the dataset was divided in a ratio of 7:2:1, resulting in a training set of 9,450 images, a validation set of 2,700 images, and a test set of 1,350 images.

### 2.2 Algorithm description of YOLOv5

In 2020, the YOLOv5 (Jocher, 2020) algorithm was officially introduced, rapidly becoming the preferred choice for mobile deployment environments due to its exceptional detection speed. YOLOv5 (Guo and Zhang, 2022) consists primarily of three components: the backbone network, the neck network, and the prediction network. The backbone network comprises a sequence of Convolutional (Conv) blocks, CSP modules, and an SPPF feature pyramid module. Its primary function is to extract feature information, translating image features into multi-level feature maps, which are subsequently transmitted to the neck network for feature fusion (Wu et al., 2019). The neck network incorporates a Feature Pyramid Network (FPN) and a Path Aggregation Network (PAN) structure; the FPN structure executes a topdown downsampling process, whereas the PAN structure conducts a bottom-up upsampling process. This architecture facilitates the fusion of feature maps across various levels, thereby enhancing the precision of object detection. The prediction network assumes a pivotal role in the initial stages of the model training process. Initially, it generates object prediction bounding boxes via the K-means clustering method and retains the bounding box with the highest confidence using the non-maximum suppression approach. Subsequently, it employs regression to ascertain the position and dimensions of the object. Finally, it categorizes each bounding box utilizing a Fully Connected (FC) layer and a softmax activation function to determine the presence of an object, while also calculating the loss value for each target box using the CIoU loss function.

**Figure 3:**
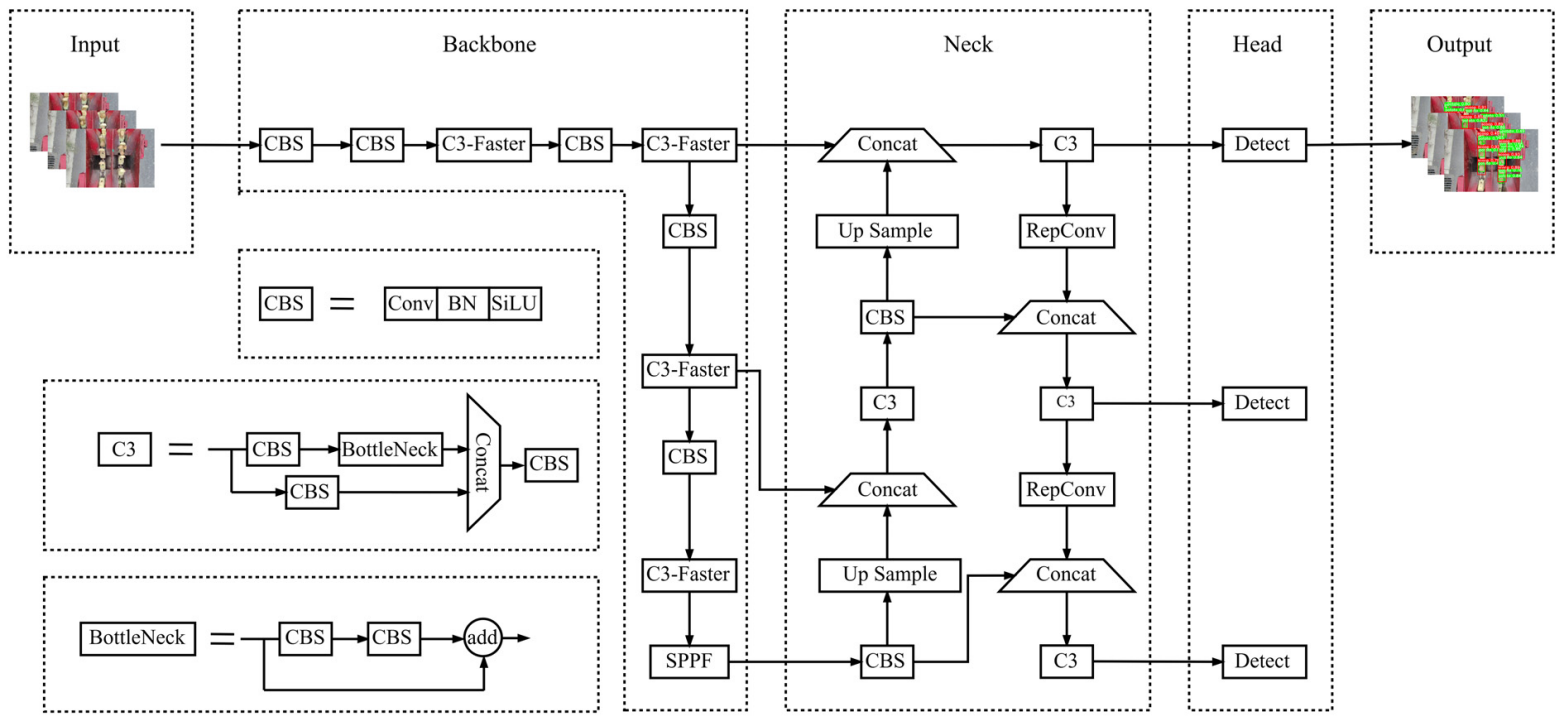
Overall structural diagram of improved YOLOv5n.

YOLOv5 version 6.0 offers five distinct model variants, namely YOLOv5n, YOLOv5s, YOLOv5m, YOLOv5l, and YOLOv5x, with the primary differences lying in the width and depth of the detection network. Each version features unique parameter configurations. In practical applications, the deployment of neural network models is often constrained by factors such as model size, memory requirements, and computational complexity. Due to the necessity of deploying the findings on embedded devices with limited computational capabilities and the requirement for real-time detection, the smallest and narrowest feature map width model, YOLOv5n, was selected as the base model for lightweight modifications. **Figure 3** illustrates the network structure of the improved YOLOv5n model. As illustrated in **Figure 3**, the key enhancements involve the substitution of the C3 and CBS modules, culminating in LAMP pruning of the refined model.

### 2.3 Improvements in network structure

#### 2.3.1 C3-Faster module

**Figure 4:**
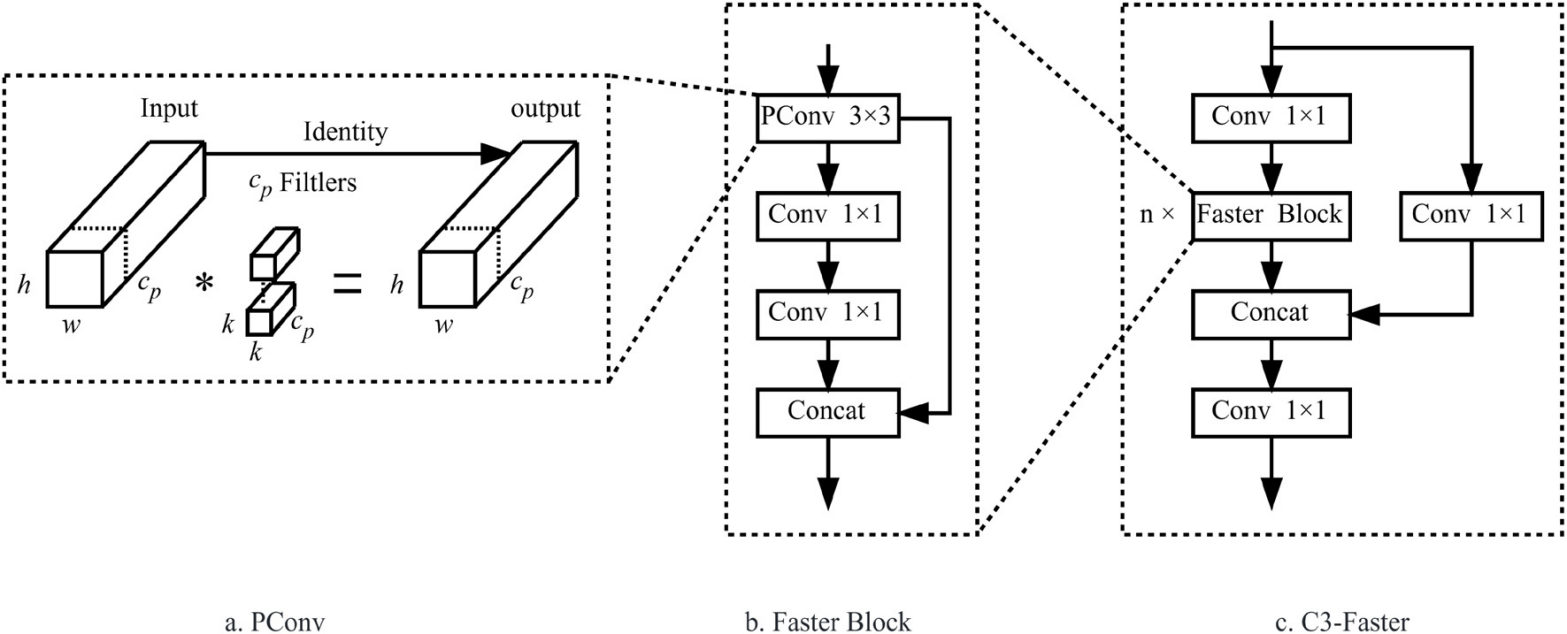
Structure diagram of PConv, FasterBlock and C3-Faster. **(a)** PConv; **(b)** FasterBlock; **(c)** C3-Faster.

Considering the memory and computational resource constraints of edge devices, the goal is to enable lightweight deployment of the model without sacrificing performance. To address this, we propose replacing the traditional C3 module in the YOLOv5 architecture with the C3-Faster module. The C3-Faster module is inspired by the PConv layer introduced in FasterNet (Chen et al., 2023), which aims to improve computational efficiency by minimizing redundant computations and memory accesses, thereby enhancing spatial feature extraction. As shown in **Figure 4a**, this approach not only reduces the overall computational burden but also optimizes memory usage, making it well-suited for resource-constrained environments.

As illustrated in **Figure 4c**, the C3-Faster structure consists of a main branch and several sub-branches. The main branch first adjusts the dimensions of the feature map through a convolutional layer, followed by feature extraction performed by multiple FasterBlocks. These FasterBlocks are then connected to the sub-branches. The output feature map is further refined through an additional convolutional layer. Importantly, the FasterBlock, shown in **Figure 4b**, consists of a PConv layer and two convolutional layers, which work together to enhance both performance and training efficiency. The key innovation of the FasterBlock lies in the use of a Shortcut mechanism, which reuses input features, thereby improving the model’s ability to efficiently capture and propagate spatial information.

#### 2.3.2 RepConv module

The feature extraction network incorporates the Reparameterizable Convolution (RepConv) module (Ding et al., 2021), which represents a novel approach to convolutional neural network design. RepConv is specifically aimed at addressing the computational complexity and memory consumption challenges commonly encountered in conventional convolutional networks. By leveraging reparameterization techniques, RepConv significantly reduces computational overhead and memory utilization, making it more efficient and suitable for deployment in resource-constrained environments, such as edge devices and real-time systems.

**Figure 5:**
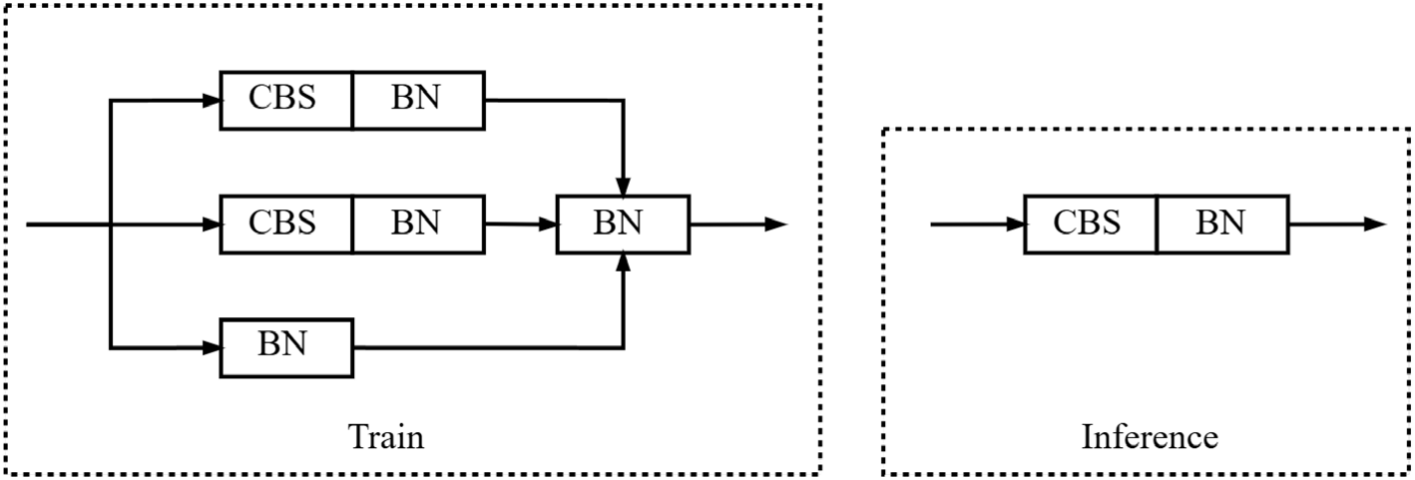
Structure diagram of RepConv.

The key advantage of RepConv lies in its ability to share parameters and increase the number of convolutional layers without the typical computational cost associated with additional layers. This is achieved through a process known as dynamic kernel reconfiguration, where the convolutional kernels are adjusted during inference to reduce the number of operations required. This dynamic flexibility allows the module to adapt its architecture based on the specific requirements of the task, enhancing its versatility across different network architectures. During the training phase, RepConv manifests as a multi-branch module; however, during the inference phase, these multi-branch modules are effectively transformed into a single-path module, with the structural details depicted in **Figure 5**.

By reparameterizing the convolutional layers, RepConv reduces the number of operations during inference. This results in faster processing times, which is especially beneficial for real-time applications or devices with limited computational resources.

#### 2.3.3 LAMP pruning

LAMP (Layer-Adaptive Magnitude-Based Pruning) is an innovative neural network pruning strategy (Lee et al., 2020), which merges global pruning significance scores and introduces the LAMP score. This scoring mechanism is pivotal in the pruning process, deciding the retention of channel structures. Currently, the prevalent evaluation criterion in pruning involves comparing the absolute values of weights, with a preference for pruning weights with smaller absolute values, deemed to contribute less to the function. The LAMP method considers both weight magnitudes and model-level distortion, obviating the need for hyperparameter tuning. In various image classification tasks, LAMP exhibits superior performance compared to traditional pruning methods.

While preserving generality, each weight tensor is transformed into a one-dimensional vector. For each flattened vector, the weights are arranged in ascending order according to the specified indexes. Let *u*, *v* denote the indexes of the weights, such that the condition $\left|W_{\left[u\right]}|\leq |W_{\left[v\right]}\right|$ is satisfied when u≤v . Subsequently, the definition of the LAMP score for $W_{\left[u\right]}$ is expressed as:


(1) $$\text{score }(u;W)=\frac{{\left(W_{\left[u\right]}\right)}^{2}}{\sum_{v\geq u}{\left(W_{\left[v\right]}\right)}^{2}}$$

(2) $${\left(W_{\left[u\right]}\right)}^{2}>{\left(W_{\left[v\right]}\right)}^{2}\Rightarrow \text{score }(u;W)>\text{score }(v;W)$$

In Equation 1, ${\left(W_{\left[u\right]}\right)}^{2}$ represents the square of the weight magnitude of the target connection, and $\sum_{v\geq u}{\left(W_{\left[v\right]}\right)}^{2}$ signifies the summation of the squares of the weight magnitudes for indexes *v* ≥ *u* within the same layer. Specifically, the denominator is defined as the cumulative sum of the squares of all connections with greater weight magnitudes in the layer, initiating from the current target index *u* (where weight terms with indexes less than *u* have been pruned). This implies the relative significance of the current connection within the layer in comparison to other connections. According to Equation 2, connections with larger weights have smaller denominators and larger numerators and therefore correspond to higher LAMP scores. This shows that the LAMP score is closely related to the weight term and the importance of the channel, and weight terms with low LAMP scores are regarded as relatively unimportant and pruned. In addition, since each layer has at least one connection with a score of 1, which is the maximum possible LAMP score, the phenomenon of layer collapse is avoided, effectively blending the advantages of global pruning and local pruning. After setting the target pruning ratio, LAMP selects the connection with the minimum LAMP score for pruning and automatically determines the layer sparsity until the global sparsity constraint is satisfied. Due to the unique design of Equations 1, 2, this process is equivalent to performing global pruning using automatically selected layer sparsity and ensures that at least one connection is retained in each layer.

This computational design facilitates the LAMP score’s efficacy as a superior metric for assessing relative importance, readily attainable through fundamental tensor operations, obviating the need for hyperparameter adjustments.

### 2.4 ByteTrack for multi-target tracking

To enable the tracking and counting of potato seed tubers and seed scoops, the Bytetrack algorithm was employed. Bytetrack is an object detection-based tracking method, which primarily relies on a data association strategy known as Byte. Unlike conventional approaches that simply discard low-confidence detections, Bytetrack differentiates between low- and high-confidence detection boxes by leveraging their respective confidence scores, and subsequently applies distinct processing strategies for each category.

**Figure 6:**
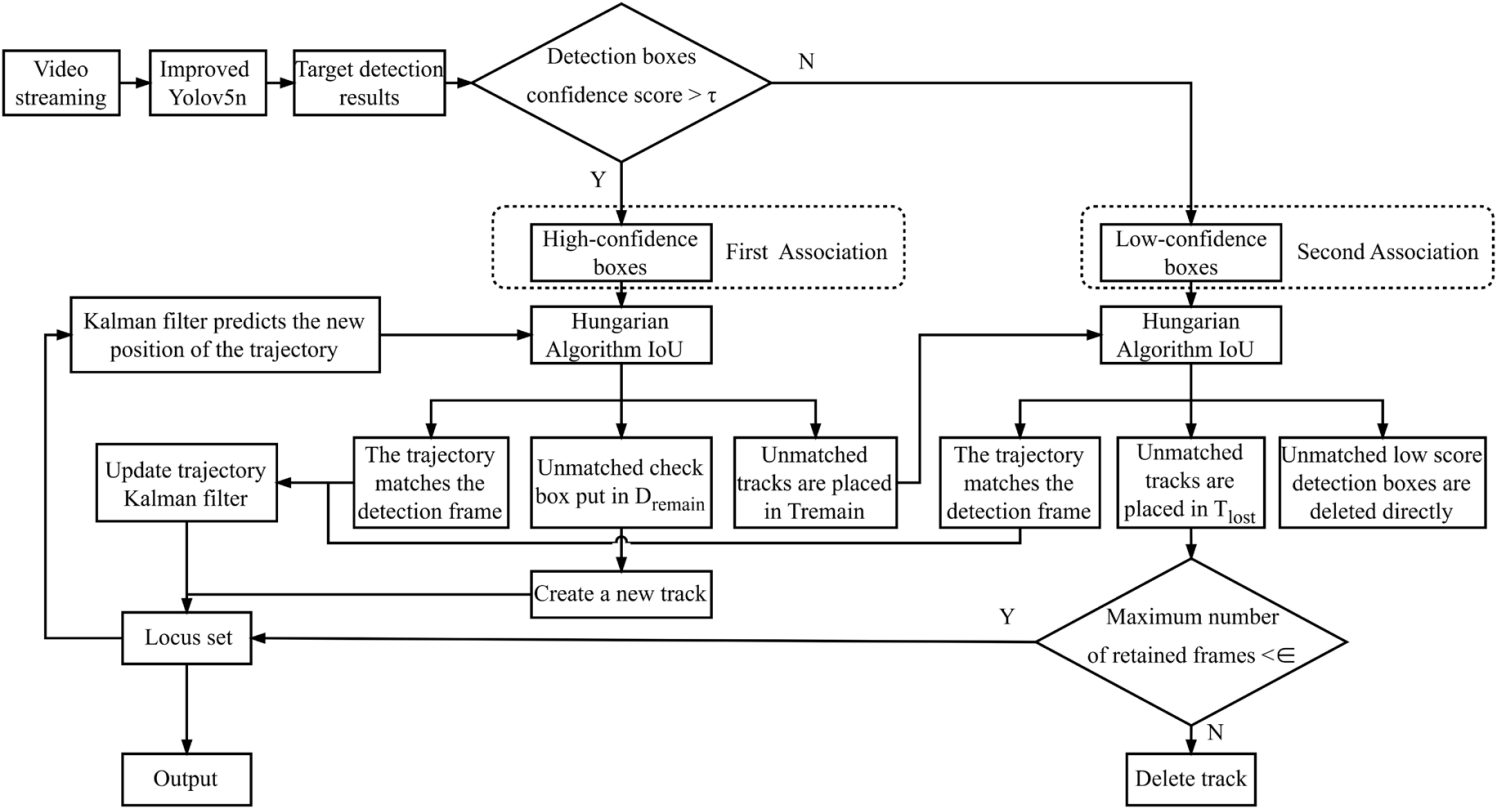
Improved YOLOv5n with Bytetrack tracking algorithm flowchart.

The Bytetrack algorithm is integrated with an improved YOLOv5n model, as depicted in the tracking system structure shown in **Figure 6**. In this framework, the improved YOLOv5n model is tasked with detecting objects within video frames and relaying the detection results to the Bytetrack algorithm. Bytetrack employs a confidence threshold to classify the detection results into high-confidence and low-confidence bounding boxes, from which trajectories are subsequently derived. Initially, high-confidence bounding boxes are correlated with existing trajectories based on the Intersection over Union (IoU) metric, which acts as the primary similarity measure, thereby optimizing the matching process. High-confidence bounding boxes that remain unmatched are utilized to initiate new trajectories, while unmatched high-confidence trajectories are preserved. A subsequent matching phase ensues, where low-confidence bounding boxes are paired with previously unmatched high-confidence trajectories, with any remaining unmatched trajectories maintained. This process facilitates the Bytetrack algorithm in assigning unique identity labels and class labels to potato seed tubers and seed scoops appearing within the video frames.

### 2.5 Counting method

The Bytetrack algorithm was employed for the purpose of tracking potato seed tubers and seed scoops. However, the operation of the potato planter may lead to alterations in the detected target IDs, meaning that the high-scoring detection frames in the current frame may not be effectively matched to the previously assigned trackers. This could potentially affect the accuracy of the counting process. To address this issue, instead of solely relying on the IDs generated by the Bytetrack algorithm for enumeration, a line-drawing based counting method was developed.

**Figure 7:**
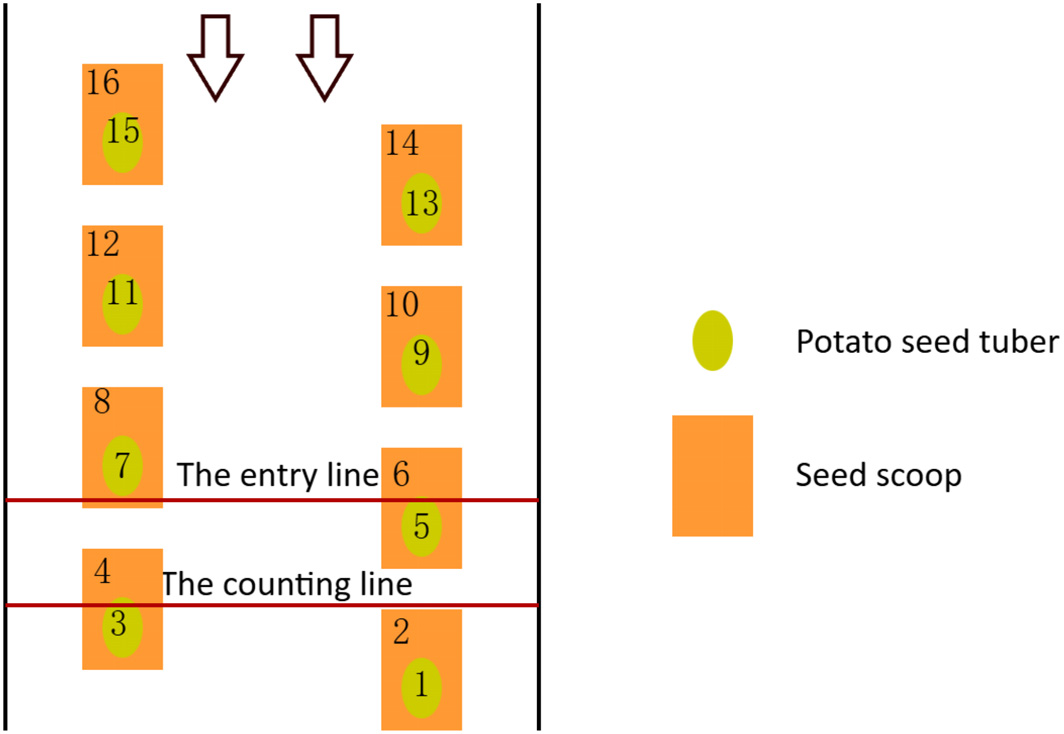
Counting method. The number indicates the tracking ID of the target, the arrow indicates that the target is moving from top to bottom.

Two lines of fixed width and position are set in the middle of the video window as shown in **Figure 7**, one as the entry line and the other as the counting line. Observed from the left part of the seed taking mechanism (the same for the right part), as the potato seed tubers and seed scoops move from top to bottom, when the center point of the target moves to the entry line, the target ID will be added to the List Left list if neither of them appears in the List Left and List Sum lists. When the target continues to move downward and its center point crosses the count line, the target ID is checked at this point to see if it is in both the List Left list and the List Sum list. If the target ID exists in the List Left list and is not in the List Sum list, the target is counted and the target ID is added to the List Sum list. The counting method for the right side is the same as for the left side, based on the position of the target’s center coordinates. If the target is located in the left side area, it is counted in the left side; if it is located in the right side area, it is counted in the right side. The counting process of the left and right sides is carried out independently at the same moment without interfering with each other. Finally, according to the changes in the number of categories in the left and right parts, the number of missed sowing, the number of replanting and the number of qualified sowing in the potato sowing process are counted, which provide a basis for the calculation of the subsequent index data. This counting method ensures that the target count is somewhat stable and accurate.

### 2.6 Evaluation metrics

To evaluate the performance of the improved model, we used metrics such as precision, recall, average precision mean(*mAP*), number of parameters, giga floating point operations per second (GFLOPs),and model size. The pertinent equations are delineated in (Equation 3, Equation 4, Equation 5, Equation 6):


(3) $$Precision=\frac{TP}{TP+FP}$$

(4) $$Recall=\frac{TP}{TP+FN}$$

(5) $$AP=\int_{0}^{1}P(R)dR$$

(6) $$mAP=\frac{1}{n}\sum_{i=1}^{n}AP_{i}$$

When potato seed tuber is categorized as a positive class and seed scoop is categorized as a negative class, True Positives (TP) are instances where the model correctly predicts potato seed tuber as a positive class; False Negatives (FN) are instances where potato seed tuber is actually a potato seed tuber but was incorrectly predicted as a negative category; False Positives (FP) are instances where the model incorrectly predicted seed scoop as a positive category; and True Negatives (TN) are instances where the model correctly predicted seed scoop as a negative category. AP is the P-R curve of a single class. The variable *n* refers to the count of target classes to be detected, whereas *mAP* signifies the mean Average Precision across all categories. Precision quantifies the ratio of correct predictions by the proposed model to the total number of predictions made. *mAP* is predominantly utilized for evaluating recognition efficacy and is extensively used in the assessment of detection model performance. *mAP*@0.5 represents the mean Average Precision computed at an Intersection over Union (IoU) threshold of 0.5. In contrast, *mAP*@0.5:0.95 denotes the *mAP* calculated across a spectrum of IoU thresholds from 0.5 to 0.95, incremented by steps of 0.05. This metric considers the model’s performance across varying degrees of overlap, thereby offering a thorough evaluation of the model’s capability to localize targets.

## 3 Results and analysis

### 3.1 Experimental Details

The hardware configuration for the model training platform includes a CPU model i5-13400, 16GB of operational memory, and a GPU RTX 3090 with 24GB of video memory. The software environment comprises a Windows 11 operating system (64-bit), Python version 3.8, the PyTorch framework version 1.10.0, and Cuda version 11.3. This paper utilizes the following parameter and hyperparameter configurations for training the YOLOv5n+C3-Faster+RepConv model: the training duration is set to 200 epochs, the initial learning rate is 0.01, the batch size is 32, the input image size is 640 × 640 pixels, the weight decay is 0.0005, momentum is 0.8, the category loss coefficient is 0.5, the bounding box loss coefficient is 0.05, the scaling ratio is 0.5, and the mosaic augmentation ratio is 1.0. The LAMP pruning strategy was applied to the model with the following training parameters: an speedup ratio of 2.0, a maximum sparsity of 1.0, 200 iterative steps, a regularization strength of 0.0005, a regularization variation of 0.0001, a training duration of 200 epochs, and a batch size of 32.

Model migration deployment embedded device: iTOP-RK3568 (TOPEET), configured with A55 quadcore ARM CPU, NPU 0.8T arithmetic power, 4G of running memory, 32G storage. Measurement system running environment: Linux system, cross-compiler compiled OpenCV, FFMPEG library.

### 3.2 Ablation study

In order to verify the effectiveness of the proposed improvement method on the performance of the YOLOv5n model, YOLOv5n is used as a baseline model, and the performance of the model is verified by adding different modules, and the experimental results are shown in **Table 1**, where the “✓” mark indicates that the corresponding improvement strategy is adopted, and the “×” indicates that it is not adopted.

**Table 1:** Ablation experiment results.

Test No.	Baseline	C3-Faster	RepConv	LAMP	mAP@0.5/%	mAP@0.5:0.95/%	Parameters	GFLOPs	Model Size/MB	Embedded Device Inference Time/ms
1	✓	×	×	×	97.3	80.6	1761871	4.1	3.7	46
2	✓	✓	×	×	98.0	81.2	1593439	3.6	3.5	41
3	✓	×	✓	×	97.7	79.9	1619055	4.2	3.9	43
4	✓	×	×	✓	95.2	78.1	852429	2.1	2.0	34
5	✓	✓	✓	×	97.7	80.9	1614495	3.7	3.6	39
6	✓	✓	✓	✓	98.0	80.8	761602	1.8	1.8	29

**Figure 8:**
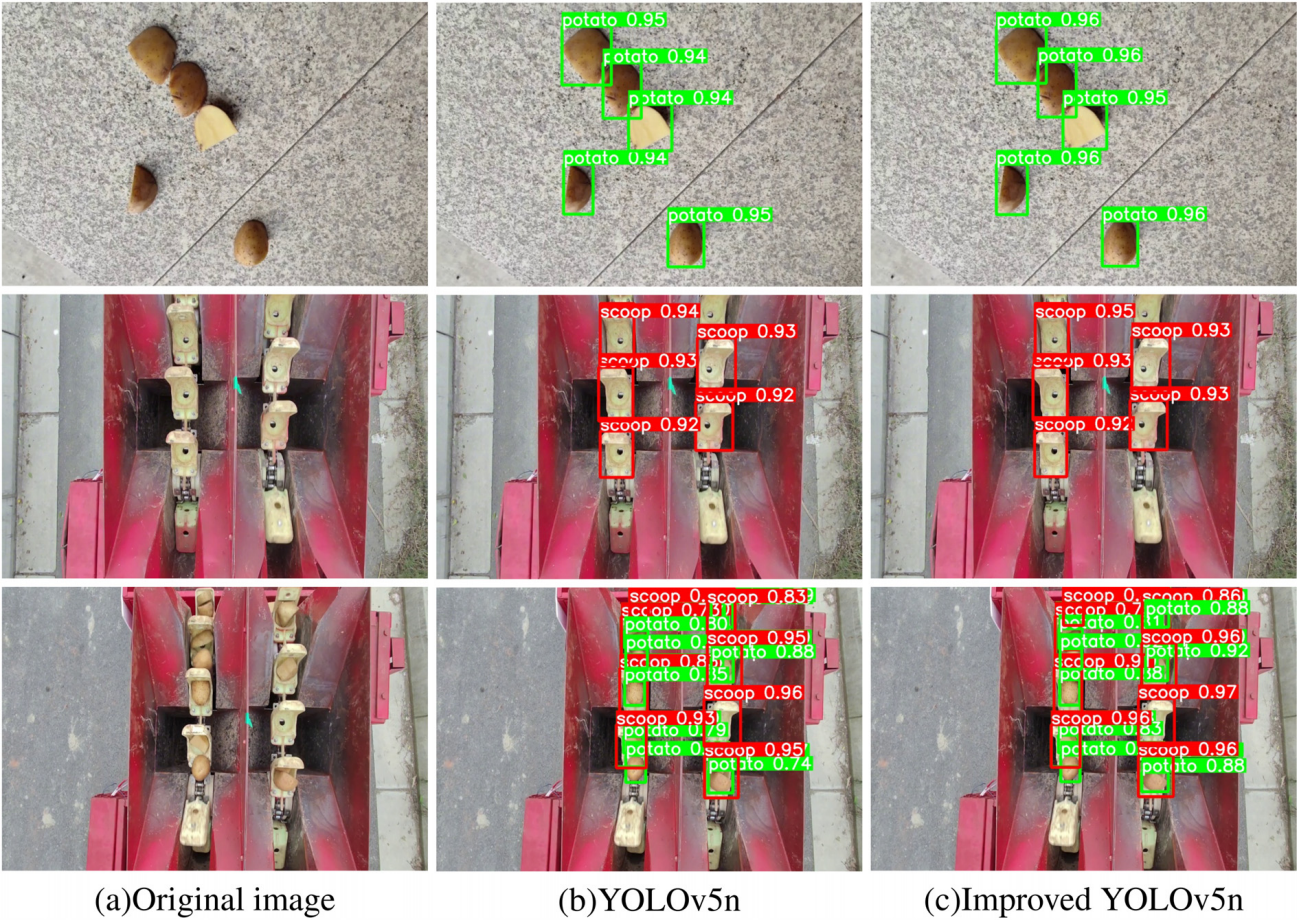
Results of model validation. **(a)** Original image; **(b)** YOLOv5n; **(c)** Improved YOLOv5n.

In Experiment 2 (**Table 1**), it is evident that the introduction of the C3-Faster module improves the model’s mAP@0.5 and mAP@0.5:0.95 while reducing the Parameters, GFLOPs, Model Size, and Embedded Device Inference Time. These results demonstrate that incorporating the C3-Faster module into FasterNet enhances the model’s operational speed. Experiment 3 shows that the inclusion of the RepConv module reduces the Parameters and Embedded Device Inference Time, confirming that RepConv contributes to faster inference. The results of Experiment 4 indicate that although the LAMP pruning technique significantly affects model accuracy, it has proven highly effective in reducing the Parameters, GFLOPs, Model Size, and Embedded Device Inference Time. Specifically, the LAMP pruning technique reduces the YOLOv5n model’s Parameters by 51.6%, GFLOPs by 48.8%, Model Size by 45.9%, and Embedded Device Inference Time by 26.1%. Despite these reductions, the mAP@0.5 only decreases by 2.1 percentage points, and the mAP@0.5:0.95 decreases by just 2.5 percentage points. To further enhance inference speed and model compression, the combination of the C3-Faster module, RepConv module, and LAMP pruning technique was explored. The results of Experiment 6 demonstrate a significant improvement in detection accuracy. Compared to the baseline YOLOv5n model, the improved model shows a 0.7 percentage point increase in mAP@0.5 and a 0.2 percentage point increase in mAP@0.5:0.95. Additionally, the model’s Parameters were reduced by 56.8%, GFLOPs by 56.1%, Model Size by 51.4%, and Embedded Device Inference Time by 37.0%. These results underscore the model’s exceptional performance in terms of parameter efficiency, computational efficacy, and lightweight design. The validation outcomes of the model are depicted in **Figure 8**.

### 3.3 Comparison of YOLO series model performance

To further evaluate the performance of the model, we compared the improved YOLOv5n model with other models in the YOLO series (Bochkovskiy et al., 2020; Wang et al., 2023; Jocher et al., 2023; Wang et al., 2024b, a), with the results shown in **Table 2**. **Table 2** shows that, although the improved YOLOv5n model slightly underperforms in terms of Precision, Recall, mAP@0.5, and mAP@0.5:0.95, it excels in Parameters, GFLOPs, and Model Size. Compared to YOLOv4-tiny, YOLOv5n, YOLOv7-tiny, YOLOv8n, YOLOv9-T, YOLOv10n and YOLOv11n, the improved YOLOv5n model reduces Parameters by 87.2%, 56.8%, 87.3%, 74.7%, 70.9%, 71.7%, and 70.5%, respectively; GFLOPs by 85.1%, 56.1%, 86.1%, 77.8%, 83.2%, 78.0%, and 71.4% respectively; and Model Size by 92.0%, 51.4%, 84.6%, 70.0%, 69.0%, 67.3%, and 65.4%, respectively. These experimental results indicate that, although the improved YOLOv5n model does not outperform some of the advanced models in terms of Precision, Recall, mAP@0.5, and mAP@0.5:0.95, its significant reductions in Parameters, GFLOPs, and Model Size make it an ideal choice for deploying high-performance object detection systems on embedded platforms.

**Table 2:** Detection results of YOLO series model.

Model	Precision /%	Recall/%	mAP@0.5/%	mAP@0.5:0.95/%	Parameters	GFLOPs	Model Size/MB
YOLOv4-tiny	95.8	96.5	97.3	80.7	5939804	12.1	22.5
YOLOv5n	96.9	95.7	97.3	80.6	1761871	4.1	3.7
YOLOv7-tiny	96.3	97.2	98.9	82.3	6010302	13.0	11.7
YOLOv8n	96.4	96.7	98.6	84.5	3006038	8.1	6.0
YOLOv9-T	96.6	97.6	99.2	84.3	2617340	10.7	5.8
YOLOv10n	97.6	97.6	99.2	84.3	2695196	8.2	5.5
YOLOv11n	97.7	97.8	99.2	85.9	2582542	6.3	5.2
Improved YOLOv5n	96.2	96.2	98.0	80.8	761602	1.8	1.8

### 3.4 Comparison of counting results

Furthermore, by integrating the improved YOLOv5n model with the Bytetrack algorithm, we have successfully achieved real-time detection, tracking, and enumeration of potato seed tubers and seed scoops using the line drawing counting technique. To validate the method’s effectiveness, this study randomly chose three video clips, each with a resolution of 640×480, from the test dataset. These clips all depicted the seed picking process of the potato planter and had undergone manual counting for verification. The results obtained from the algorithm were then compared to the manual counts, and the pertinent data are presented in **Table 3**. The experimental results showed that seed scoop with regular shape and large target reached 100% counting accuracy, but potato seed tubers with small target and irregular shape failed to reach 100% counting accuracy. For the average accuracy of target counting, 96.6% accuracy can meet the requirement of potato precision planter metering system.

**Table 3:** Comparison of counting results.

Test No.	Category	Manual counting	Algorithmic counting	Accuracy/%
1	seed scoops	61	61	100.0
	potato seed tubers	72	67	93.1
2	seed scoops	48	48	100.0
	potato seed tubers	57	53	93.0
3	seed scoops	67	67	100.0
	potato seed tubers	77	73	94.8
Average		382	369	96.6

### 3.5 Potato precision planter metering system

Qt is a cross-platform C++ application framework widely recognized for its superior efficiency, scalability, and cross-platform compatibility, making it a popular choice for visual interface development. In this study, a potato precision planter metering system was designed and developed using Qt version 5.0, integrating an improved YOLOv5n model, the ByteTrack algorithm, and a counting method. Through cross-compilation, the system was successfully deployed on the development board, enabling precise metering of potato seed tubers and seed scoops.

**Figure 9:**
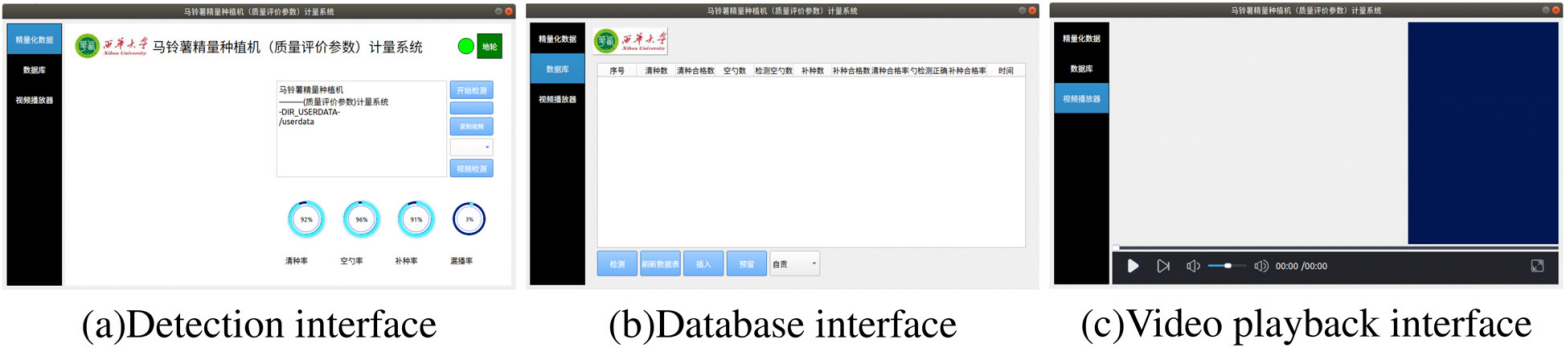
Interface of the metering system. **(a)** Detection interface; **(b)** Database interface; **(c)** Video playback interface.

The potato precision planter metering system consists of three parts: the detection interface, the database interface, and the video player interface, as shown in **Figure 9**. The detection interface is used for real-time monitoring and display of potato planting results; the database interface is responsible for data storage and query functions; the video player interface is responsible for playing user-recorded videos.

**Figure 10:**
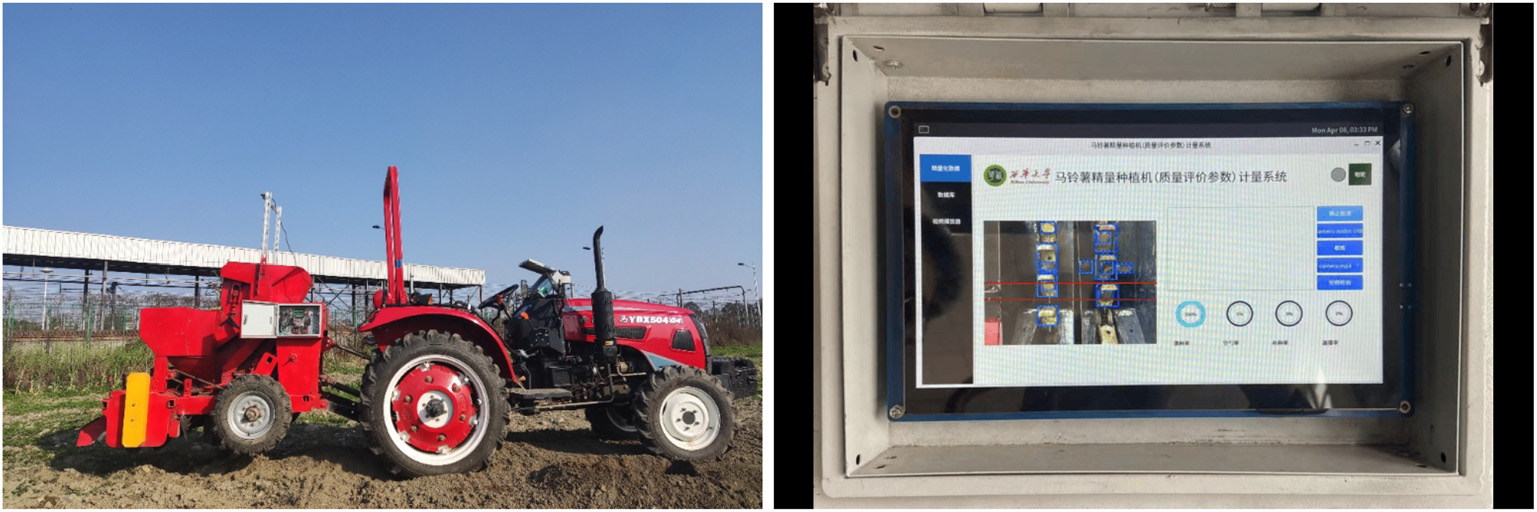
Potato precision planter metering system.

As shown in **Figure 10**, we mounted the camera on top of the seed clearing device to capture the image of the seed clearing position at a top-down angle. At the same time, the camera was connected to the iTOP-RK3568 development board in the box in front of the tractor’s steering wheel via a USB harness. At this point, the potato precision planter metering system has been successfully integrated into the potato precision planter. Once the system is activated, the camera will capture the data of the seed picking process in real time and present it instantly in the designated display area of the inspection interface.

## 4 Discussion

As a globally important food crop, potato plays a crucial role in solving the global hunger problem (Wang and Su, 2024). With the development of smart agriculture, deep learning techniques, especially YOLO series, ResNet, CNN and LSTM, have been widely used in potato production. These techniques can significantly improve yield and economic efficiency, especially in tasks such as potato bud eye recognition, leaf disease diagnosis and health monitoring. Currently, the assessment of potato planting effect still relies on the traditional manual method, which is labor-intensive and costly. Therefore, the development of an efficient and lightweight detection model suitable for embedded devices is the key to improving work efficiency.

Although there are excellent algorithms for detection in potato production such as Xiu et al. (Xiu and Sun, 2022) proposed an automatic potato seedling bud detection method based on YOLOv4 network. Zhang et al. (2023) proposed an improved potato sprout detection algorithm based on YOLOv5s. In addition, Liu et al. (2024a) proposed an improved Bud-YOLOv8s model. Experimental results show that this model performs well in the potato eye detection task, and therefore greatly outperforms other models in the YOLO family. However, fast detection in potato production is still challenging, especially the balanced detection performance on embedded devices with limited computational power, where the speed and model parameters are not yet satisfactory.

To address this problem, in this paper, based on engineering experience and experimental results, we compare and analyze the YOLO series of algorithms for target detection. We choose YOLOv5n as the base network and improve it in lightweight. As a result, we proposed a potato planting machine’s seed potato scooping scene detection algorithm based on the improved lightweight YOLOv5n model, and combined it with the ByteTrack algorithm and counting method to successfully develop a metering system for real-time monitoring of the planting effect of potato precision planters.

Although the improved YOLOv5n has demonstrated excellent detection performance and detection speed in inspection tasks, there are still some limitations. The performance of the potato precision planter metering system is affected by a number of factors during practical application. Due to the performance of the embedded devices, the accuracy of target tracking tends to decrease when the potato planter is traveling too fast, resulting in poor performance of the potato precision planter metering system. In addition, lighting conditions (excessive or insufficient) can also affect the accuracy of the potato precision planter metering system in detecting seed potato nuggets and picking scoops.

In future work, we will further expand the dataset and enrich the images with various scenes and lighting conditions to improve the robustness of the model. To address the problem of degradation of tracking accuracy due to the high speed of potato planters, we plan to use higher performance embedded devices and USB industrial cameras with high frame rates without significantly increasing the hardware cost. In addition, we note the recent progress of YOLOv10 and YOLOv11. We will integrate state-of-the-art target detection algorithms to further optimize the system performance in our future work, thus improving the stability and accuracy of detection and counting, and further improving the performance of the potato precision planter metering system.

## 5 Conclusions

With the rapid advancement of deep learning, object detection and tracking algorithms have been widely applied in agriculture. This study addresses the challenge of accurately evaluating potato planting performance by integrating existing agricultural object recognition and counting techniques, aiming to improve data acquisition in mechanized potato planting. To this end, a detection algorithm for potato seeding scenes was proposed, based on an improved lightweight YOLOv5n model. The model was optimized by incorporating the C3-Faster and RepConv modules while employing LAMP pruning to enhance its efficiency. To enable precise counting, the ByteTrack algorithm was introduced, along with a dedicated counting method tailored for potato seeding scenes. Ultimately, a metering system for precision potato planters was developed, combining the improved YOLOv5n model, ByteTrack algorithm, and counting method to facilitate accurate assessment of potato planting effectiveness. The experimental results show that, compared to the baseline YOLOv5n model, the improved YOLOv5n model demonstrated superior performance in detecting potato seed tubers and seed scoops. Specifically, the improved model showed a 0.7 percentage point increase in mAP@0.5 and a 0.2 percentage point increase in mAP@0.5:0.95. Additionally, the model reduced parameters by 56.8%, GFLOPs by 56.1%, model size by 51.4%, and Embedded Device Inference Time by 37.0%. When compared to mainstream YOLO series models, the improved YOLOv5n model exhibited comparable performance in Precision, Recall, mAP@0.5, and mAP@0.5:0.95, while significantly reducing the number of parameters, GFLOPs, and model size. Furthermore, the proposed counting method effectively detected both potato seed tubers and seed scoops, achieving a counting accuracy of 96.6%, thus meeting the application requirements for the metering system. The study offers technical support for assessing the planting performance of potato planters. Potato precision planter metering system is capable of evaluating the effectiveness of potato planting and promoting high-quality development of the potato planting industry.

## Data Availability

The raw data supporting the conclusions of this article will be made available by the authors, without undue reservation.
